# Cardiac Structure and Function of Elite Australian Jockeys Compared to the General Population: An Observational Cross-Sectional Study

**DOI:** 10.1186/s40798-024-00783-9

**Published:** 2024-11-05

**Authors:** Angela Farley, Hunter Bennett, Roger Eston, Rebecca Perry

**Affiliations:** Alliance for Research in Exercise, Nutrition and Activity (ARENA), UniSA Allied Health & Human Performance, University of South Australia, City East Campus, Frome Road, Adelaide, South Australia 5001 Australia

**Keywords:** Thoroughbred horse racing, Jockey, Athlete’s heart, Echocardiography, 2D echocardiography

## Abstract

**Background:**

Research highlights the intense physiological demands of thoroughbred racing on jockeys, with elevated heart rates and substantial oxygen uptake, confirming the rigorous physical nature of the sport, however, the cardiovascular changes resulting from the physical demands of thoroughbred racing remain unexplored in Australian jockeys. Therefore, the objective of this study was to compare measures of cardiac structure and function of professional Australian jockeys to that of the general population and to determine if there are differences in heart structure and function detected using echocardiography.

**Methods:**

Forty-six jockeys and thirty-three participants from the general population underwent two-dimensional echocardiography, which included all standard views and measurements. Each measurement was compared between groups using a Mann-Whitney U test.

**Results:**

Groups were matched for age (jockeys (35 ± 12 years) and controls (36 ± 13 years)). Jockeys were shorter (1.64 ± 0.07 m vs. 1.75 ± 0.09 m, *p* < 0.001), lighter (56.5 ± 6.0 kg vs. 74.2 ± 12.9 kg, *p* < 0.001) and had a lower body surface area (BSA) (1.55 ± 0.17 m^2^ vs.1.9 ± 0.2 m^2^, *p* < 0.001). Jockeys had a larger absolute left ventricular (LV) end diastolic volume than the control group (120 ± 18.2 ml vs. 109.3 ± 29.0 ml, *p* = 0.05) which had a larger variation when indexed for BSA (78.0 ± 12.2 ml/m^2^ vs. 57.5 ± 13.3 ml/m^2^, *p* < 0.001). Jockeys demonstrated a higher LV mass index (79.4 ± 18.1 g/m^2^ vs. 64.2 ± 15.4 g/m^2^, *p* < 0.001). Left atrial volume index was larger in jockeys (33.4 ± 6.5 mL/m^2^ vs. 26.3 ± 7.0 mL/m^2^, *p* < 0.001). There were no differences in global longitudinal strain (GLS) for either group overall (-19.3 ± 3.0% vs. -19.8 ± 1.6%, *p* = 0.52), but 17% of the jockey group demonstrated an abnormal GLS.

**Conclusions:**

Jockeys have adaptations to their cardiac structure and function compared to the general population. Differences could be attributed to chronic physiological demands of racing and should be considered in future research involving jockeys.

## Key Points

- Jockeys exhibit distinctive cardiac adaptations that are comparable to other athlete groups, including larger LV and LA volumes, and LV mass when compared to the general population.
- These adaptations are likely attributed to the chronic physiological and physical demands of horse racing.
- Further research should explore whether these changes increase with time spent participating in the sport.

## Background

Thoroughbred horse racing is one of the oldest and most lucrative sports in the world [1]. Jockeys are at the forefront of this dynamic and at times perilous competition with their performance having a direct impact on race outcomes [2]. In Australia, there are approximately 844 registered jockeys participating in over 19,000 races annually [3]. Race riding demands physical strength, stamina, and quick decision-making during races [4]. Although there are many studies on the race fitness required by equine athletes, there is little data around jockey participants [4].

A literature review of the physiological demands of racing conducted by Ryan and Brodine (2021) identified only three studies that measured heart rate and mean oxygen uptake (VO_2_) of jockeys during race conditions. It was demonstrated that jockeys experience elevated heart rates throughout each race (130–180 beats/min) with peak heart rates ranging from 150 to 190 beats/min [5–7]. Substantial oxygen uptake was also demonstrated during races with oxygen uptake values ranging from 42.7 (± 5.6) to 57.5 (± 4.7) mlO_2_/kg/min [5–7]. These findings indicate the rigorous physical nature of the sport and demonstrate the need for endurance (isotonic) fitness for this group of athletes [5–7].

On top of endurance (isotonic) fitness, the unique crouched posture that jockeys maintain during races requires strength (isometric) fitness [4]. This posture, often referred to as the “martini glass” position, involves jockeys standing in the stirrups while keeping their knees bent and their bodies forward and parallel to the horse’s body [3]. They must also stay low and close to the horse’s neck [3]. This position ensures better aerodynamics and helps jockeys to absorb the horse’s movements through their legs [3].

Drawing from studies utilizing skin surface electromyography (EMG) and Training Impulse (TRIMP) scores, (used to quantify the load or intensity of an athlete’s training over time and originally defined as the product of training volume, measured in minutes, and training intensity, normally measured as average heart rate) to quantify the muscle activity used by jockeys while maintaining this unique squat posture, it is clear that jockeys experience isometric exercise loads comparable to elite athletes in other sports such as soccer players [4]. In a study by Legg et al. (2022) the TRIMP score for jockeys during 16 professional races was quantified at 292 ± 106 arbitrary units (au) [4]. This is noteworthy considering the TRIMP experienced by a professional soccer player during a 1.5-hour match approximates ~ 190 au, whereas that of a world-class marathon runner participating in a 2-hour race reaches approximately 275 au [4]. These findings highlight the quasi-isometric muscle activation that occurs during racing and therefore the athletic requirements while maintaining this position, indicating that jockeys require a unique blend of both endurance and strength fitness [4], and suggest they may experience cardiovascular changes in line with other athletic populations.

Cardiovascular physiological adaptations can occur due to the unique demands of regular and intense physical activity in the hearts of athletes [8]. Adaptations commonly seen due to chronic and high-intensity exercise include enlargement of all four cardiac chambers, an increase in left ventricular (LV) wall thickness, and enhanced diastolic filling of the LV [9]. This remodelling allows for increased stroke volume (SV) which in turn increases cardiac output when exercising [9]. Whilst these changes have been well demonstrated in many elite athlete groups such as rowers and cyclists [10], the cardiovascular changes resulting from the physical demands of thoroughbred racing remain unexplored in this population of athletes.

The physiological demands of this sport on jockey participants extend further than mere athleticism. Beyond the elite fitness requirements previously mentioned, jockeys face the additional challenge of maintaining a low body mass (50–61 kg) to meet race-related weight requirements [6]. Often this is daily and, as there is no ‘off season’, many attempt to ‘cut weight’ year-round [11]. Weight-loss techniques, including jogging in sweat gear, hot baths, and saunas, coupled severe energy restriction and dehydration, are commonly used by jockeys to maintain a chronically low body mass [5]. There is evidence to suggest that chronic food and fluid restriction can have detrimental effects on cardiac structure and function in the general population as seen in patients with Anorexia Nervosa (AN) [12]. These practices can lead to decreased LV chamber size, left atrial (LA) enlargement, impaired myocardial contractility, altered diastolic function, reduced stroke volume, and decreased cardiac output [12]. Additionally, changes in Global Longitudinal Strain (GLS) may indicate impaired myocardial performance [12]. The combination of these adaptations can potentially increase the risk of cardiac dysfunction, arrhythmias, and other long-term cardiovascular complications [12–14]. However, to the authors’ knowledge, this has yet to be investigated in jockeys.

Therefore, the aim of this study was to determine if there are differences in heart structure and function detected using echocardiography in registered Australian jockeys when compared to the general population. We hypothesised that cardiac remodelling related to athletic training, such as increased cardiac chamber sizes and greater LV mass, would be demonstrated in jockeys when compared to the general population.

## Materials and methods

### Participant Selection

Professional Australian jockeys holding a current riding license in South Australia and Western Australia were invited to take part in the study. Data were collected from all jockeys on days of rest from race riding. Participants from the general population were invited using social media advertisements. Whilst there were no restrictions on physical activity for participants recruited to the study, they were excluded if they were < 18 years of age or if there was a previously undocumented heart abnormality discovered during the echocardiogram. All participants gave written informed consent, and the study was approved by the University of South Australia Human Research Ethics Committee (project number: 204235).

### Anthropometric Measurements

Immediately prior to their echocardiogram, anthropometric data were collected from all participants. Height (with shoes and socks removed) was assessed to the nearest centimetre using a portable stadiometer (Seca 213, Hamburg, Germany). Body mass and Total body water % (TBW %) were measured in minimal light clothing using portable digital weighing scales (Tanita Innerscan, Body Composition Monitor, BC-541). Body Surface Area (BSA) was calculated according to the formula of Dubois and Dubois as a ratio of weight (kg) to height (m) [15].

### Echocardiography

Two-dimensional (2D) echocardiography included all standard views and measurements. These were taken in accordance with American Society of Echocardiography guidelines [16]. All images were acquired from parasternal and apical windows with participants in the left lateral decubitus position and imaging was completed by one member of the research group, a qualified echocardiographer. Commercially available echocardiography systems with either a M55c XD clear Matrix or 4Vc phased array transducer were used for image acquisition (GE E95, Vivid IQ, General Electric, Horten, Norway). Images were recorded digitally in cine-loop format. Advanced image analysis was performed using commercially available analysis software (TOMTEC Image Arena and Autostrain analysis, Unterschleissheim, Germany). As part of a standard echocardiogram, specific images for diastolic function including mitral valve inflow and tissue Doppler imaging (TDI) were taken. Left atrial volume (LAV) was measured from both the apical 4 and 2 chamber views with care to open the LA to its major axis. LV volumes and ejection fraction were measured using the Simpson’s rule of discs from the apical 4 and 2 chamber views where the major axis of the LV was found. Stoke volume was calculated from the LV outflow tract diameter and velocity time integral. LV GLS was calculated from LV focused images from the apical 4-chamber, 2-chamber, and long-axis views. Studies were deemed inadequate for GLS analysis if 2 or more LV segments could not be visualized. A region of interest was automatically applied by the software to the endocardial and epicardial borders, with tracking adjusted to cover the entire myocardium. GLS was calculated as the change in the length of line of the region of interest across all apical view from diastole to systole. To determine intra-rater reliability, the echocardiographic data from 20 jockeys and 20 normal controls was analysed twice with a minimum duration of one month between measurements. Intra-operator variability was determined using intraclass correlation coefficients (ICC) with an ICC < 0.5 indicating poor agreement, 0.5 to 0.7 moderate agreement and > 0.8 good agreement [17].

### Lifestyle Questionnaire

Jockey participants were also required to complete a lifestyle questionnaire modified from a previous study [18]. The questionnaire collected information on years of jockeying, race riding statistics and weekly exercise activities completed in addition to race riding. Information on previous medical history was also obtained. Where applicable, participants were instructed to select the most appropriate descriptor, select as many options as deemed personally relevant or type extra information. The survey tool Qualtrics (XM, Provo, UT) was used to design and distribute the questionnaire and to record responses.

### Statistical Analysis

This study was reported in accordance with the Strengthening the Reporting of Observational studies in Epidemiology (STROBE) guidelines.

All statistical analyses were conducted using Statistical Package for the Social Sciences (SPSS) (v26, IBM, USA) with the level of statistical significance set at *p* < 0.05. Parametric data were presented as mean ± SD and nonparametric data as median (interquartile range) unless otherwise stated. A Mann-Witney U test to determine differences between the specific groups, jockeys, and general population, was used as the data were not normally distributed. Cohen’s effect size [ES] was calculated to determine differences between the two groups with the difference between the means divided by the pooled standard deviation calculated. Effect sizes were calculated (= z /sqrt(N)) and interpreted as follows: <0.20 = *trivial*, 0.20–0.49 = *small*, 0.50–0.79 = *moderate*, and > 0.79 = *large* [19].

Furthermore, as BSA has been suggested to be flawed for indexing cardiac parameters in very small or large people [20], an exploratory analysis was conducted to identify whether any significant differences could be explained by differences in body size, whereby 19 variables were compared between the eight smallest participants in the control group and eight largest participants in the jockey groups using the above statistical methods.

As the groups were not sex matched, and this is a known area of variation in terms of cardiac remodelling [10], subgroup analysis was also performed comparing the females in the jockey group to the females in the control group. Males in the jockey group were also compared with the males in the control group.

Sample size was calculated using the normal value of GLS (-20 ± 3%) and the expectation of a 10% clinically relevant change in GLS between the normal population and jockeys. With reference to Diggle et al., (2002) this means that a sample size of 13 participants in both groups was required for 80% power to detect a clinically significant difference between jockeys and controls with a significance level (α) of 0.05 [21]. To account for possible participant dropout, we aimed to recruit a minimum of 20 participants per group.

## Results

Forty-six Australian jockeys (age range: 18–51 years of age) and thirty-three participants from the general population (control group) (age range:18–52 years of age) consented to take part in this study. Descriptive and mean anthropometric data for the jockey and control groups are presented in Table 1. There were no significant differences in age (*p* = 0.54) and sex (*p* = 0.11) between the jockeys and the control group. Jockeys were shorter (*p* < 0.001), weighed less (*p* < 0.001) and therefore had a lower BSA than the control group (*p* < 0.001). Total Body Water % (TBW %) was recorded for the jockey group only. As were minutes of physical activity per week which included aerobic activity not related to race riding.

**Table 1:** Descriptive data (mean ± SD) for controls (*N* = 33) and jockeys (*N* = 46)

Value	Control (*N* = 33)	Jockey (*N* = 46)	U =	*p*-Value	Effect size
Age (years)	36 ± 13	35 ± 12	697.50	0.54	0.07
Sex (males %)	17 (52%)	32 (70%)	622.0	0.11	0.19
Weight (kg)	74.2 ± 12.9	56.5 ± 6.0*	119.0	**< 0.001**	0.73
Height (m)	1.75 ± 0.09	1.64 ± 0.07*	252.5	**< 0.001**	0.58
BSA (m^2^)	1.9 ± 0.2	1.55 ± 0.17*	118.0	**< 0.001**	0.73
TBW (%)	Not recorded	59.3 ± 4.4	N/A	N/A	N/A
Minutes physical activity per week (mins)	Not recorded	1182 ± 414	N/A	N/A	N/A

*p < 0.05 compared with control group: U - Mann-Witney U test value, BSA – Body surface area, TBW – Total body water %, Values are presented as mean ± standard deviation or number and (percentage). Bold text indicates significant p-values

**Fig. 1:**
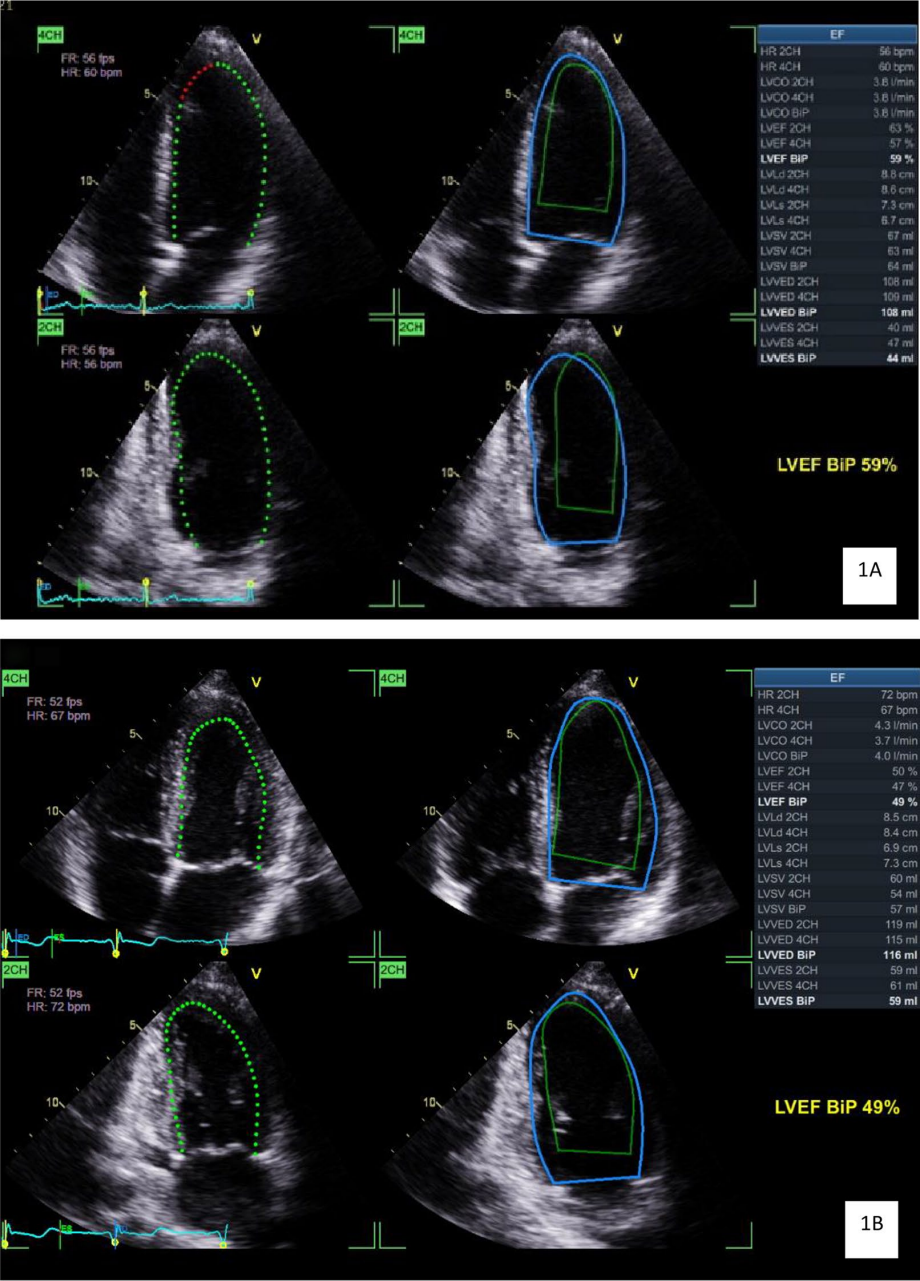
1A and 1B: Echocardiographic images demonstrating the measurement of left ventricular volume and ejection fraction from a general population participant (**A**) demonstrating a left ventricular end diastolic volume of 109mL and ejection fraction of 59% and in a jockey (**B**) demonstrating a larger left ventricular end diastolic volume of 116mL and lower ejection fraction of 49%. The dotted green line represents the instantaneous volume, the solid blue line represents end diastolic volume, and the solid green line represents end systolic volume. Abbreviations: 2CH – two chamber view, 4CH – four chamber view, BiP – biplane, bpm – beats per minute, cm – centimetres, CO- cardiac output, EF – ejection fraction, HR – heart rate, L – litres, LV – left ventricular, ml – millilitres, VED – volume at end diastole, VES – volume at end systole

The results of the echocardiographic measurements for the jockey and control groups are presented in Table 2. Even though their height, weight and BSA were less than the control group, jockeys had a larger LV end diastolic volume (LVEDV) (*p* = 0.05), which remained when indexed for BSA (*p* < 0.001) and higher LV ejection fractions (LVEF) (*p* < 0.001). Example images representing the comparison of group averages of these values can be seen in Fig. 1A and B.

Absolute LV mass did not differ between the two groups (*p* = 0.92); however, the jockey group demonstrated a higher LV mass index than the control group (*p* < 0.001).

**Fig. 2:**
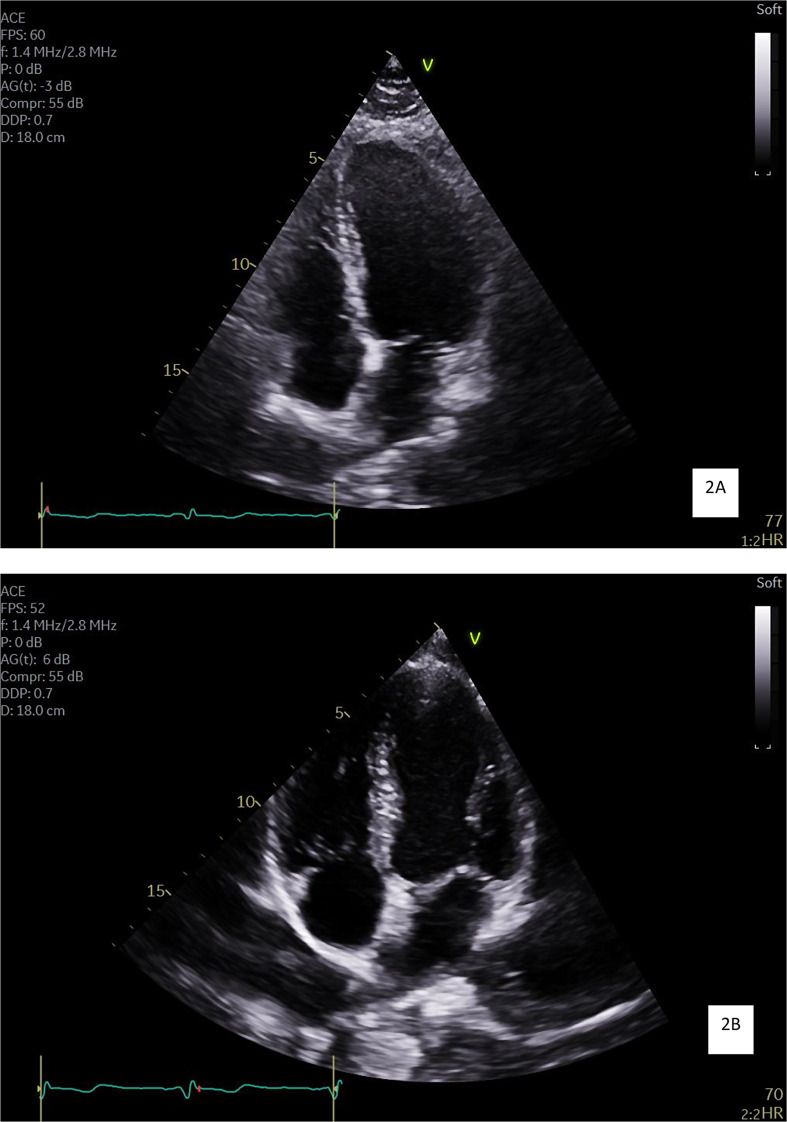
2A and 2B: Apical 4-chamber echocardiogram images demonstrating the chambers sizes in a general population participant (**A**) and jockey (**B**). Chronic high intensity physical activity results in dilation of cardiac chambers, particularly the left ventricle [22]

Furthermore, LAV values were larger in jockeys, but only when indexed for BSA (*p* < 0.001). Example images representing the comparison of group averages of the chamber sizes can be seen in Fig. 2A and B.

A lower stroke volume (SV) (*p* = 0.021) was also demonstrated by the jockey group. However, when SV was indexed for BSA there was no longer a difference between the groups (*p* = 0.32).

**Table 2:** Echocardiographic measurements (mean ± SD) for controls (*N* = 33) and jockeys (*N* = 46)

Value	Control (*N* = 33)	Jockey (*N* = 46)	U =	*p*-Value	Effect size
HR (bpm)	60 ± 11	65 ± 13	577.5	0.23	0.14
GLS (%)	-19.8 ± 1.6 (-17 to -23%)	-19.3 ± 3.0 (-11 to -25%)	695.0	0.52	0.07
LVEDV (mL)	109.3 ± 29.0	120.0 ± 18.2	547.0	0.06	0.22
LVESV (mL)	47.4 ± 13.6	48.7 ± 12.1	710.5	0.8	0.03
LVEF (%)	57 ± 3	60.8 ± 5.2*	386.0	**< 0.001**	0.41
Mitral E: A ratio	1.9 ± 0.6	2.1 ± 0.9	654.5	0.415	0.09
E: E’ (avg)	5.6 ± 1.2	6.2 ± 1.5	556.0	0.09	0.19
Stroke Volume (mL)	75.7 ± 20.7	64.1 ± 12.6*	473.0	**0.02**	0.26
Stroke Volume index (mL/m^2^)	39.8 ± 9.3	41.7 ± 8.2	601.0	0.32	0.11
Cardiac Output (L/min)	4.5 ± 1.4	4.1 ± 0.9	592.0	0.298	0.12
LA vol (biplane, mL)	49.7 ± 13.8	51.6 ± 10.7	593.5	0.341	0.11
LA vol index (mL/m^2^)	26.3 ± 7.0	33.4 ± 6.5*	311.0	**< 0.001**	0.46
LVEDV index (mL/m^2^)	57.5 ± 13.3	78.0 ± 12.2*	195.0	**< 0.001**	0.63
LV mass (g)	124.2 ± 35.3	123.8 ± 36.7	748.5	0.917	0.01
LV mass index (g/m^2^)	64.2 ± 15.4	79.4 ± 18.1*	429.0	**< 0.001**	0.34

*p < 0.05 compared with control group: U - Mann-Witney U test value, HR – heart rate, GLS – global longitudinal strain, LVEDV – left ventricular end diastolic volume, LVESV – left ventricular end systolic volume, LVEF – left ventricular ejection fraction, Mitral E: A ratio – ratio of peak velocity blood flow from left ventricular relaxation in early diastole to peak velocity flow in late diastole, E:E’ – mitral inflow early diastolic velocity to tissue Doppler mitral annular early diastolic velocity ratio, LA vol – left atrial volume, LV – left ventricular, Values are presented as mean ± standard deviation or number and (percentage). Bold text indicates significant p-values

Despite the GLS being similar for both groups, there were 8 (17%) jockeys who had a GLS more impaired than − 16%, whereas all of the control group demonstrated normal GLS. Of these 8 jockeys, 7 had been riding for more than 5 years and 1 had been riding for less than 5 years. 5 were male and 3 were female. 1 was between the ages of 20–30, 3 were between the ages of 30–40 and 4 were between the ages of 40–50.

The eight largest jockeys and eight smallest of the control group were also compared. There was no difference in BSA between the 2 groups (*p* = 0.28) suggesting that any cardiac changes observed are independent of body size. LVEDV, LVESV, LAV, LVEDV indexed for BSA, LV mass and LV mass index were all larger in jockeys compared to the control group (*p* < 0.05).

Females in the control group and the jockey group, along with males in each group were compared. The results remained significant for weight, height, BSA, LVEDVi, LVEF and LAVi (*p* < 0.05) between groups. However, only female jockeys demonstrated a higher LV mass index compared to the female control group 74.9 ± 17.9 vs. 56.5 ± 12.7 g/m^2^, *p* = 0.004). Whereas male jockeys did not when compared to the male control group (81.3 ± 18.1 vs. 73.3 ± 13.4 g/m^2^, *p* = 0.13).

ICC of all measurements ranged from 0.85 to 0.95 (all p values < 0.0001) indicating good intra-observer agreement.

## Discussion

To the authors’ knowledge, this is the first study to evaluate the cardiac structure and function of jockey athletes, revealing distinctive cardiac adaptations of considerable interest that may be comparable to other athlete groups. A critical finding is the presence of physiological remodelling of cardiac chambers in jockeys, particularly when indexed for BSA. Notably, jockeys exhibited larger LV and LA volumes as well as increased LV mass when indexed for BSA compared to the general population.

It is crucial to emphasize that these alterations in chamber dimensions were not associated with impaired cardiac function, as evidenced by the jockeys demonstrating normal LVEF [23]. It has been demonstrated that athletes generally have LVEF values that are close to the general population [22]. As this is a critical indicator of cardiac systolic function, the preservation of normal LVEF observed in the jockey group, would suggest that despite the increased LA and LV volumes, the contractile capacity of their hearts was not compromised [24].

This observed increase in LV and LA volumes as well as LV mass, when indexed for BSA, could be attributed to the chronic high-intensity physical regimens jockeys complete [6]. According to the World Health Organisation’s 2020 physical activity guidelines, adults are advised to engage in 150–300 min of moderate intensity physical activity, 75–150 min of vigorous intensity physical activity or a combination of both, weekly [25]. Jockey participants in this study, reported performing much more exercise than these recommendations, indicating exposure to considerably elevated levels of physical activity when compared to the general population [25]. Therefore, this extensive physical activity maybe a factor for the structural and functional changes in jockeys’ hearts observed in this study. It has been well documented that an increase in cardiac chamber volumes, particularly LV, occurs in athletes exposed to high levels of physical activity, particularly those that train for endurance sports with both isometric and isotonic exercise, such as rowers and cyclists [10]. The haemodynamic overload and increased cardiac output during exercise helps to better perfuse working muscles [22]. Therefore, giving athletes a competitive edge, particularly those in endurance sports where an increase in the size of LA and RA volumes is seen [10]. Given the increase in both LA and LV volumes observed in the jockey group, this suggests that this population, a group that has not previously been included in athlete heart studies, fit within this demographic of endurance athletes [10, 26, 27]. Whilst it was not a focus of this study, this finding necessitates further research comparing jockeys to other athlete groups to understand the full extent of these adaptations.

Additionally, cardiovascular adaptions seen in athletes do not occur as discrete, all-or-nothing changes, but instead as gradual progression that is proportional with the intensity and duration of training [22]. Perry et al. (2018) examined the physiological changes in 145 Australian Football players over a 6 year period [9]. They demonstrated typical cardiac remodelling that is generally seen in athletes such as increased LV mass and volume in proportion to exercise levels with RV remodelling becoming more apparent with longevity of training and increasing age [9]. They also found that these changes occur over time and are dependent on individual fitness level, physical activity type and age [9]. Therefore, it is plausible to suggest more time spent in the sport could lead to further cardiac adaptations [22]. Thus, examining jockeys over career longevity is another possible avenue for future research.

Jockeys also displayed an increased left ventricular end-diastolic volume (LVEDV), although this was not statistically significant until it was indexed for BSA. This is a common observation in athlete hearts, similar to that seen in endurance athletes such as rowers and cyclists [10, 22, 27]. Regular intensive training, especially endurance training, leads to increased preload due to a higher blood volume and enhanced venous return [28]. This increased preload stretches the ventricular walls, promoting chamber enlargement and increased LVEDV [28]. Physiological remodelling then allows the heart to pump a greater volume of blood per beat, contributing to improved overall cardiovascular efficiency during high-intensity physical activities, such as was observed in the jockey group [28].

Jockeys exhibited significantly lower weight, height, and BSA compared to the control group. This may be attributed to the rigorous weight management requirements they face to meet racing standards, combined with the generally shorter stature of jockeys compared to the general population [29]. These factors could potentially influence cardiac parameters as, whilst their LVEF and GLS were clinically normal as a group, it is important to note that 17% (8) of the jockeys in this study presented with reduced GLS. GLS is a sensitive marker of myocardial deformation and can reveal subclinical changes in cardiac function such as LV dysfunction and hypertrophic myocardiopathy [9, 30]. It has been reported in previous studies, that no difference in GLS has been found in endurance trained rugby players, elite cyclists or rowers when compared to sedentary controls or recreational athletes [31]. This was further supported in a meta-analysis in which no athlete-control differences existed for GLS [31]. Although this study was not designed to delve deeply into specific causes, the finding that 7 of the 8 jockeys with reduced GLS had been riding for more than 5 years, may suggest that certain practices associated with this unique athlete group may lead to undesirable adaptations in cardiac function detected only by GLS. Especially as all age matched general population participants demonstrated GLS within a normal range. One avenue for future investigation may centre on the effects of weight-shedding practices commonly employed by jockeys to meet the rigorous weight requirements of their profession. These practices, which include strategies such as saunas and extreme dietary restrictions, could conceivably exert detrimental effects on myocardial function, similar to those seen in patients with the early stages of AN where altered GLS while systolic function is preserved can indicate early myocardial deformation, thus warranting further exploration [14, 32].

With this in mind, chronic dehydration and calorie deficiencies are known to demonstrate reduced chamber volumes, decreased LVEF and CO [12]. However, the jockey group demonstrated TBW (%) within a normal range for athletes [33–35]. Matais et al. (2016), examined the body composition of 200 athletes from 15 sports including basketball, track and field, soccer, wrestling, tennis, swimming and sailing, demonstrating a mean TBW (%) of ~ 50% [35]. Similar values have been observed in marathon runners (57%) and rowers (54%) [33, 34, 36]. This suggests that the jockey group in the present study were comparable to other athletes and, on the day of data collection, were not dehydrated. This could be due to the measurements being taken on a day of rest when jockeys had not been restricting their diet or sweating in preparation for race day. This also suggests that hydration did not affect preload and LVEDV values of jockeys during echocardiography data collection.

Also, given the findings for height, weight and BSA for the jockey group, it is consistent that jockeys demonstrated a lower absolute stroke volume; however, this was within normal limits and did not differ from the control group when indexed to BSA. This finding aligns with previous studies such as one conducted by De Simone et al., (1997) which observed a relationship between stroke volume and measures of body size [37]. When compared to the general population it would be expected that jockeys also have a lower absolute stroke volume [37].

### Limitations

While the study’s results are consistent with the hypothesis of cardiac remodelling in jockeys, it is important to acknowledge the limitations of the cross-sectional design. While the control group used in this study were free of cardiovascular disease, they were not required to meet any physical activity guidelines or report their exercise habits [25]. Therefore, the results may not be replicated in highly active individuals from the general population, and it is unclear whether differences in activity levels were driving the differences seen in cardiac structure and function seen between groups. Also, the sample sizes for both groups were relatively small, which may have affected the statistical power to detect significant differences in some parameters. This may limit the generalisability of these results to other jockey populations. However, the small jockey sample size can be attributed to the low number of jockeys that compete Australia wide, and therefore is a good representation of jockeys competing in Australia. Additionally, while groups were matched for age, given the exploratory nature of the research and the small sample size, they were unable to be matched for sex. There is evidence that differences in sex can influence certain parameters of the heart such as chamber size [16], which may have influenced the results. However, as the data was normalised for BSA, it is unlikely to have had a notable impact.

## Conclusions

This study investigated the cardiovascular structure and function of a group that has not previously been examined. It provides insights into the cardiovascular characteristics of registered Australian jockeys compared to the general population. The observed differences in LA volume index, LVEDV index and LV mass index between the two groups may be due to the high level of exercise experienced by the jockey participants. However, as this study was cross-sectional, it cannot be determined if this relationship was causative. These findings highlight the need for further research on this specialised athlete population, especially against other elite athletes. Also, the identification of a subset of jockeys with reduced GLS raises questions about the effects of the physiological demands of racing and is a potential area for future investigation.

## Data Availability

The datasets used and/or analysed during the current study are available from the corresponding author on reasonable request.

## Abbreviations

EMG: Electromyography
TRIMP: Training Impulse
au: Arbitrary Units
LV: Left ventricular
LA: Left atrial
GLS: Global Longitudinal Strain
AN: Anorexia Nervosa
BSA: Body Surface Area
2D: Two-dimensional
TDI: Tissue Doppler imaging
LAV: Left atrial volume
SPSS: Statistical Package for the Social Sciences
LVEDV: LV end diastolic volume
LVEF: LV ejection fraction
SV: Stroke volume
E: E’: Mitral inflow early diastolic velocity to tissue Doppler mitral annular early diastolic velocity ratio
HR: Heart rate
LVESV: Left ventricular end systolic volume
SD: Standard deviation
TBW (%): Total Body Water %
2CH: Two chamber view
4CH: Four chamber view
BiP: Biplane
bpm: Beats per minute
cm: Centimetres
CO: Cardiac output
EF: Ejection fraction
L: Litres
ml: Millilitres
VED: Volume at end diastole
VES: Volume at end systole

## References

[1] Davies M, Jackson KA, Mackinnon AL, Turner A, Kuznik K, Hill J, et al. Epidemiology of race day injury in young professional jockeys in Great Britain from 2007 to 2018: a retrospective cohort study. BMJ open. 2021;11(8):e044075–e. 34380713 10.1136/bmjopen-2020-044075 PMC8359493

[2] Hitchens PL, Hill AE, Stover SM. Jockey falls, injuries, and fatalities Associated with Thoroughbred and quarter Horse Racing in California, 2007–2011. Orthop J Sports Med. 2013;1(1):2325967113492625. 26535231 10.1177/2325967113492625 PMC4555501

[3] Giusti Gestri L. Wearable technology may assist in reducing jockeys’ injuries if integrated into their safety vests: a qualitative study. Front Sports Act Living. 2023;5:1167110. 37416317 10.3389/fspor.2023.1167110 PMC10321524

[4] Legg K, Cochrane D, Gee E, Macdermid P, Rogers C. Physiological demands and muscle activity of jockeys in Trial and Race Riding. Anim (Basel). 2022;12(18):2351. 10.3390/ani12182351 PMC9495223 36139208

[5] Cullen S, OʼLoughlin G, McGoldrick A, Smyth B, May G, Warrington GD. Physiological demands of flat Horse Racing jockeys. J Strength Cond Res. 2015;29(11):3060–6. 25932980 10.1519/JSC.0000000000000977

[6] Kiely M, Warrington GD, McGoldrick A, Pugh J, Cullen S. Physiological demands of professional flat and Jump Horse Racing. J Strength Cond Res. 2020;34(8):2173–7. 32735425 10.1519/JSC.0000000000003677

[7] O’Reilly J, Cheng HL, Poon ET-C. New insights in professional horse racing in-race heart rate data, elevated fracture risk, hydration, nutritional and lifestyle analysis of elite professional jockeys. J Sports Sci. 2017;35(5):441–8. 27070776 10.1080/02640414.2016.1171890

[8] Addis DR, Townsley MM. Imaging considerations for the athletically conditioned heart: an Echocardiography-focused overview of the 2020 American Society of Echocardiography Recommendations on the Use of Multimodality Cardiovascular Imaging in Young Adult competitive athletes. J Cardiothorac Vasc Anesth. 2020;34(11):2867–70. 32711936 10.1053/j.jvca.2020.06.077

[9] Perry R, Swan AL, Hecker T, De Pasquale CG, Selvanayagam JB, Joseph MX. The spectrum of change in the elite athlete’s heart. J Am Soc Echocardiogr. 2019;32(8):978–86. 31202591 10.1016/j.echo.2019.04.006

[10] Pelliccia A, Caselli S, Sharma S, Basso C, Bax JJ, Corrado D, et al. European Association of Preventive Cardiology (EAPC) and European Association of Cardiovascular Imaging (EACVI) joint position statement: recommendations for the indication and interpretation of cardiovascular imaging in the evaluation of the athlete’s heart. Eur Heart J. 2018;39(21):1949–. 29029207 10.1093/eurheartj/ehx532

[11] Wilson G, Chester N, Eubank M, Crighton B, Drust B, Morton JP, et al. An alternative dietary strategy to make weight while improving mood, decreasing body fat, and not dehydrating: a case study of a professional jockey. Int J Sport Nutr Exerc Metab. 2012;22(3):225–31. 22693243 10.1123/ijsnem.22.3.225

[12] Meucci M, Locorotondo G, Della Casa S, Filippo Crea M, Galiuto L. Echocardiographic Assessment of Cardiac Complications in Anorexia Nervosa: correlation with the Disease Severity and the role of global longitudinal strain. A unique case. J Gynecol Women’s Health. 2020;19(5).

[13] StÖHr EJ, Gonzalez-Alonso J, Pearson J, Low DA, Ali L, Barker H, et al. Dehydration reduces left ventricular filling at rest and during exercise independent of twist mechanics. J Appl Physiol (1985). 2011;111(3):891–7. 21700893 10.1152/japplphysiol.00528.2011

[14] Watanabe K, Stöhr EJ, Akiyama K, Watanabe S, González-Alonso J. Dehydration reduces stroke volume and cardiac output during exercise because of impaired cardiac filling and venous return, not left ventricular function. Physiol Rep. 2020;8(11):e14433-n/a. 32538549 10.14814/phy2.14433 PMC7294577

[15] Verbraecken J, Van de Heyning P, De Backer W, Van Gaal L. Body surface area in normal-weight, overweight, and obese adults. A comparison study. Metab Clin Exp. 2006;55(4):515–24. 16546483 10.1016/j.metabol.2005.11.004

[16] Lang RM, Badano LP, Mor-Avi V, Afilalo J, Armstrong A, Ernande L, et al. Recommendations for cardiac chamber quantification by echocardiography in adults: an update from the American Society of Echocardiography and the European Association of Cardiovascular Imaging. Eur Heart J Cardiovasc Imaging. 2015;16(3):233–71. 25712077 10.1093/ehjci/jev014

[17] Liljequist D, Elfving B, Skavberg Roaldsen K. Intraclass correlation - A discussion and demonstration of basic features. PLoS ONE. 2019;14(7):e0219854–e. 31329615 10.1371/journal.pone.0219854 PMC6645485

[18] Dolan E, Crabtree N, McGoldrick A, Ashley DT, McCaffrey N, Warrington GD. Weight regulation and bone mass: a comparison between professional jockeys, elite amateur boxers, and age, gender and BMI matched controls. J Bone Miner Metab. 2012;30(2):164–70. 21773703 10.1007/s00774-011-0297-1

[19] Cohen J. Statistical power analysis for the behavioral sciences. 2nd ed. ed. Hillsdale, N.J: L. Erlbaum Associates; 1988.

[20] Dewey FE, Rosenthal D, Murphy DJ, Froelicher VF, Ashley EA. Does size Matter? Clinical applications of scaling cardiac size and function for body size. Circulation. 2008;117(17):2279–87. 18443249 10.1161/CIRCULATIONAHA.107.736785

[21] Diggle P. Analysis of Longitudinal Data. Second edition. ed. United Kingdom: Oxford University Press; 2013. xv-xv p.

[22] Prior DL, La Gerche A. The athlete’s heart. Heart. 2012;98(12):947–55. 22626903 10.1136/heartjnl-2011-301329

[23] Yılmaz M, Kayançiçek H, Elevated LV, Mass. LV Mass Index sign on the Athlete’s ECG: athletes’ hearts are prone to ventricular arrhythmia. J Clin Med. 2018;7(6):122. 29843381 10.3390/jcm7060122 PMC6024950

[24] Kuchynka P, Palecek T, Vilikus Z, Havranek S, Taborska K, Louch WE, et al. Cardiac structural and functional changes in competitive amateur cyclists. Echocardiography (Mount Kisco NY). 2010;27(1):11–6. 10.1111/j.1540-8175.2009.00965.x 19765071

[25] Bull FC, Al-Ansari SS, Biddle S, Borodulin K, Buman MP, Cardon G, et al. World Health Organization 2020 guidelines on physical activity and sedentary behaviour. Br J Sports Med. 2020;54(24):1451–62. 33239350 10.1136/bjsports-2020-102955 PMC7719906

[26] Kovacs RMD, Baggish ALMD. Cardiovascular adaptation in athletes. Trends Cardiovasc Med. 2016;26(1):46–52. 25976477 10.1016/j.tcm.2015.04.003

[27] Pluim BM, Zwinderman AH, Van Der Laarse A, Van Der Wall EE. The athlete’s heart: a meta-analysis of cardiac structure and function. Volume 101. New York, NY): Circulation; 2000. pp. 336–44. 3. 10.1161/01.cir.101.3.336 10645932

[28] Boraita A, Díaz-Gonzalez L, Valenzuela PL, Heras M-E, Morales-Acuna F, Castillo-García A et al. Normative values for Sport-Specific Left Ventricular dimensions and Exercise-Induced Cardiac Remodeling in Elite Spanish Male and female athletes. Sports Med - Open. 2022;8(1). 10.1186/s40798-022-00510-2 PMC9478009 36107355

[29] Wilson G, Drust B, Morton JP, Close GL. Weight-making strategies in Professional jockeys: implications for physical and Mental Health and Well-Being. Sports Med. 2014;44(6):785–96. 24682950 10.1007/s40279-014-0169-7

[30] Abou R, van der Bijl P, Bax JJ, Delgado V. Global longitudinal strain: clinical use and prognostic implications in contemporary practice. Heart. 2020;106(18):1438–44. 32404401 10.1136/heartjnl-2019-316215

[31] Flanagan H, Cooper R, George KP, Augustine DX, Malhotra A, Paton MF, et al. The athlete’s heart: insights from echocardiography. Echo Res Pract. 2023;10(1):15. 37848973 10.1186/s44156-023-00027-8 PMC10583359

[32] Morris R, Prasad A, Asaro J, Guzman M, Sanders L, Hauck A, et al. Markers of Cardiovascular Dysfunction in adolescents with Anorexia Nervosa. Glob Pediatr Health. 2017;4:X233379417727423–2333794. 10.1177/2333794X17727423 PMC5580842 28890913

[33] Kendall KL, Fukuda DH, Hyde PN, Smith-Ryan AE, Moon JR, Stout JR. Estimating fat-free mass in elite-level male rowers: a four-compartment model validation of laboratory and field methods. J Sports Sci. 2017;35(7):624–33. 27159216 10.1080/02640414.2016.1183802

[34] Sebastiá-Rico J, Soriano JM, González-Gálvez N, Martínez-Sanz JM. Body composition of male Professional Soccer players using different measurement methods: a systematic review and Meta-analysis. Nutrients. 2023;15(5):1160. 36904159 10.3390/nu15051160 PMC10005265

[35] Matias CN, Santos DA, Júdice PB, Magalhães JP, Minderico CS, Fields DA, et al. Estimation of total body water and extracellular water with bioimpedance in athletes: a need for athlete-specific prediction models. Clin Nutr. 2016;35(2):468–74. 25886709 10.1016/j.clnu.2015.03.013

[36] Tam N, Nolte HW, Noakes TD. Changes in total body water content during running races of 21.1 km and 56 km in athletes drinking ad libitum. Clin J Sport Med. 2011;21(3):218–25. 21427566 10.1097/JSM.0b013e31820eb8d7

[37] De Simone G, Devereux RB, Daniels SR, Mureddu G, Roman MJ, Kimball TR, et al. Stroke volume and cardiac output in normotensive children and adults: Assessment of relations with body size and impact of overweight. Volume 95. New York, NY: Circulation; 1997. pp. 1837–43. 7. 10.1161/01.cir.95.7.1837 9107171

[38] World Medical Association Declaration of Helsinki. Ethical principles for medical research involving human subjects. Taehan Ŭisa Hyŏphoe Chi. 2014;57(11):899. 25951678

